# Platelets Enter the Itch Circuit

**DOI:** 10.34133/research.1381

**Published:** 2026-07-21

**Authors:** David Stegner

**Affiliations:** ^1^ Institute of Experimental Biomedicine, Chair I, University Hospital Würzburg, 97080 Würzburg, Germany.; ^2^ Rudolf Virchow Center for Integrative and Translational Bioimaging, Julius-Maximilians University Würzburg, 97080 Würzburg, Germany.

Chronic itch is increasingly understood not merely as a symptom of skin inflammation, but as the result of active neuroimmune communication within inflamed barrier tissues. Sensory neurons, immune cells, stromal cells, and vascular components interact in local circuits that can sustain both inflammation and pruritus. In this issue of *Research*, Hu and colleagues [1] add an unexpected cellular player to this circuitry: the platelet.

Platelets have long been viewed primarily through the lens of hemostasis and thrombosis. Over the past decade, however, this perspective has progressively broadened. Beyond sealing vascular injury, platelets are now recognized as dynamic inflammatory effector cells that shape leukocyte recruitment, vascular integrity, innate immune activation, and tissue injury across a wide spectrum of diseases [2]. Emerging evidence further suggests that platelets participate in inflammatory processes within barrier tissues such as the skin, where platelet activation has been linked to chronic inflammatory disorders, including psoriasis and atopic dermatitis [3–5].

The study by Hu and colleagues expands upon this concept by positioning platelets directly within the neuroimmune circuitry of inflamed skin. Using optogenetic platelet activation, transcriptomic analyses, neuronal recordings, pharmacological interventions, and genetic mouse models, the authors provide evidence that platelet activation is not merely associated with cutaneous inflammation and itch, but can actively drive both through a platelet–immune–neuron axis [1].

Conceptually, the work connects 3 biological processes often considered separately: vascular perturbation, immune-cell recruitment, and sensory-neuron sensitization. In inflamed skin, vascular activation and altered barrier function may favor platelet recruitment and activation. Activated platelets then release vasoactive and inflammatory mediators that promote macrophage accumulation and shape the local immune milieu. These platelet-derived mediators, or secondary signals generated by recruited immune and stromal cells, may sensitize TRPV1+ pruriceptors and sustain C-fiber hyperexcitability. By selectively activating platelets in vivo, Hu and colleagues demonstrate that platelet stimulation alone is sufficient to induce inflammatory skin changes, macrophage accumulation, and scratching behavior [1]. Thus, platelets emerge as a dynamic link between vascular disturbance, immune amplification, and itch-associated neuronal signaling.

Mechanistically, serotonin emerges as one important mediator within this axis. Platelet-derived serotonin has long been implicated in vascular and inflammatory biology, but its role in chronic skin inflammation has remained less well-defined. Here, pharmacological and genetic approaches implicate serotonergic signaling via HTR2B in TRPV1+ pruriceptors as a contributor to platelet-driven itch responses [1]. Importantly, platelet-derived mediators should not be viewed only as acute neuronal activators. They may also lower sensory thresholds or enhance the expression and function of ion channels and receptors involved in itch signaling [6], thereby promoting sustained C-fiber hyperexcitability. At the same time, the broader implications likely extend beyond serotonin alone. Platelets contain chemokines, cytokines, lipid mediators, growth factors, and immunomodulatory molecules capable of influencing immune, stromal, endothelial, and neuronal cell populations [2]. The work therefore raises the possibility that platelets may coordinate inflammatory sensory microenvironments and longer-lasting sensory sensitization.

These findings align with clinical observations linking platelet activation to inflammatory skin disease. In patients with atopic dermatitis or psoriasis, increased plasma levels of the platelet activation markers β-thromboglobulin and platelet factor 4 have been reported, with correlations with disease severity scores and reduction after clinical improvement [3,5,7]. Additional studies of psoriasis have reported elevated platelet-derived microparticles and soluble P-selectin, further supporting systemic platelet activation in inflammatory skin disease. More recently, platelets have been recognized as active contributors to cutaneous immune regulation and autoinflammatory processes rather than passive bystanders of inflammation [4,8]. The current study adds mechanistic depth to these clinical associations by connecting platelet activation with sensory neuronal activation and itch behavior [1].

The work also fits into a broader shift in platelet biology. Platelets are increasingly viewed as mobile inflammatory sentinels capable of translating vascular perturbations into local immune responses [2]. Recent work on platelet-derived integrin- and tetraspanin-enriched tethers has further underscored that platelets can amplify inflammatory tissue injury independently of classical thrombus formation [9]. Together with the study by Hu and colleagues, these findings challenge a strict separation between thrombosis, immunity, and tissue-specific inflammatory biology.

This perspective may also extend beyond the skin. Chronic inflammatory skin diseases are increasingly recognized as disorders with systemic immune and vascular components. As circulating immune effector cells, platelets are well-suited to carry or amplify inflammatory cues from barrier tissues through platelet–leukocyte aggregates, soluble mediators, microparticles, and endothelial interactions in distant vascular beds [10]. Although direct proof for a platelet-mediated skin-to-organ relay remains limited, such a mechanism could help connect chronic cutaneous inflammation with systemic immune alterations and organ-level dysfunction in tissues such as the lung, spleen, liver, or brain.

At the same time, the study raises important conceptual questions. One concerns the spatial relationship between platelets, immune cells, and sensory neurons within inflamed skin. Hu and colleagues report increased vascular permeability and vasodilation, providing a plausible structural context through which platelets can access perivascular or extravascular inflammatory niches [1]. Nevertheless, whether platelets remain largely intravascular, localize to perivascular regions, translocate across altered vascular barriers, or exploit leukocyte-driven changes in vascular permeability requires further investigation. Equally important is whether platelet–neuron communication occurs through direct spatial proximity or through intermediary cell types such as macrophages, mast cells, keratinocytes, endothelial cells, or stromal cells.

Additional questions concern the broader role of platelets in chronic inflammatory disease. Are platelets primary initiators of neuroimmune inflammation, or do they mainly amplify preexisting inflammatory circuits? To what extent are platelet–neuron interactions relevant beyond the skin, for example, in pulmonary, intestinal, or neuroinflammatory disorders? Which platelet-derived mediators cooperate with serotonin to shape sensory responses, and how tissue-specific are these mechanisms? Addressing these questions will require spatially resolved and cell-type-specific approaches capable of mapping platelet localization, mediator release, and cellular communication in vivo. Such studies will be particularly important to define where platelets reside in inflamed skin and how HTR2B and HTR7 signaling is distributed across TRPV1+ pruriceptors, macrophages, endothelial cells, and other inflammatory or stromal cell populations.

From a translational perspective, the study raises the possibility that selected platelet-derived mediators, rather than general platelet activation, could be targeted to modulate chronic itch and inflammation while avoiding unwanted interference with hemostasis. This distinction may be important, as platelets retain essential vascular protective functions even in inflammatory settings. A more precise understanding of platelet-derived neuroimmune signals could therefore open therapeutic opportunities that uncouple pathological sensory inflammation from core hemostatic functions.

By placing platelets directly into the itch circuit, Hu and colleagues extend the conceptual boundaries of platelet biology. Their study suggests that platelets are not only vascular sentinels and inflammatory amplifiers, but may also shape sensory neuronal activity in chronically inflamed tissues. Thus, the work once again expands the functional repertoire of platelets, adding neuroimmune communication to their growing list of activities in inflammation and tissue biology.
